# 3D Markerless asymmetry analysis in the management of adolescent idiopathic scoliosis

**DOI:** 10.1186/s12891-018-2303-4

**Published:** 2018-10-24

**Authors:** Maliheh Ghaneei, Amin Komeili, Yong Li, Eric C. Parent, Samer Adeeb

**Affiliations:** 1 Department of Civil and Environmental Engineering, Donadeo-ICE, University of Alberta, 9203 116th St, Edmonton, AB T6G 1R1 Canada; 2 Faculty of Kinesiology, University of Calgary, Calgary, Canada; 3 Department of Physical Therapy, Faculty of Rehabilitation Medicine, University of Alberta, Edmonton, Canada

**Keywords:** Scoliosis, Surface topography, 3D markerless asymmetry analysis, Monitoring, Curve progression

## Abstract

**Background:**

Three dimensional (3D) markerless asymmetry analysis was developed to assess and monitor the scoliotic curve. While the developed surface topography (ST) indices demonstrated a strong correlation with the *Cobb angle* and its change over time, it was reported that the method requires an expert for monitoring the procedure to prevent misclassification for some patients. Therefore, this study aimed at improving the user-independence level of the previously developed 3D markerless asymmetry analysis implementing a new asymmetry threshold without compromising its accuracy in identifying the progressive scoliotic curves.

**Methods:**

A retrospective study was conducted on 128 patients with Adolescent Idiopathic Scoliosis (AIS), with baseline and follow-up radiograph and surface topography assessments. The suggested “cut point” which was used to separate the deformed surfaces of the torso from the undeformed regions, automatically generated deviation patches corresponding to scoliotic curves for all analyzed surface topography scans.

**Results:**

By changing the “cut point” in the asymmetry analysis for monitoring scoliotic curves progression, the sensitivity for identifying curve progression was increased from 68 to 75%, while the specificity was decreased from 74 to 59%, compared with the original method with different “cut point”.

**Conclusions:**

These results lead to a more conservative approach in monitoring of scoliotic curves in clinical applications; smaller number of radiographs would be saved, however the risk of having non-measured curves with progression would be decreased.

## Background

Adolescent idiopathic scoliosis (AIS) is the most common form of three-dimensional (3D) spinal deformity. It affects 2–4% of the population, predominantly females [1]. The AIS spine deformity progresses rapidly during the adolescent growth period, resulting in a need for frequent follow-ups [2]. The gold standard for assessing the spine curve is measuring the *Cobb angle* on the full torso radiograph, defined as the angle between the two most tilted vertebrae in each curve [3].

The conventional monitoring of the scoliosis using the *Cobb Angle* has limitations recognized in the literature. Firstly, the measurement is limited to 2D posterior-anterior radiographs, and thus the method fails to address the 3D characteristics of AIS [4]. In addition, the use of radiographs in scoliosis clinics has several pitfalls of growing concern, such as excessive X-ray radiation exposure, with their associated risk of developing cancers [5–9], and the contra-indication of radiograph acquisitions for pregnant women.

Surface topography (ST) was introduced as a new approach to improve the monitoring of the scoliosis [5, 10–12]. ST is a method for which non-invasive visible light is used for scanning the torso surface in order to assess cosmetic deformities often based on some landmarks placed on the patient’s torso by trained clinicians and using related indices based on the coordinates of the landmarks with respect to each other and the geometric properties of transverse cross section of the torso, such as the cosmetic score and Quantec spinal angle [13–17]. Measurements based on ST could possibly be used effectively in combination with the radiographs to decrease the radiation dose and risk of cancer resulting from the multiple X-ray acquisitions. At this point, the ST approach is by no means designed to fully replace the gold-standard radiograph measurements, because it is subject to validation with the radiograph measurements. Nevertheless, the development of accurate ST methods has significantly contributed to the management of scoliosis [12].

On the other hand, marker placements can be associated with human errors in collecting the raw data [14] and such methods fail to take into account the whole torso geometry in the analysis. In contrast, our team has developed a novel markerless ST asymmetry analysis approach, which is independent of human interactions, and considers the full 3D torso surface for the analysis [12]. This technique provides a deviation contour map that visualizes the areas affected by AIS corresponding to the location of each curve, called deviation patch [12]. These patches are isolated to calculate ST parameters for such deviations. The method demonstrated the potential for reducing 44% of radiograph exposure in the monitoring of scoliosis [15].

**Fig. 1:**
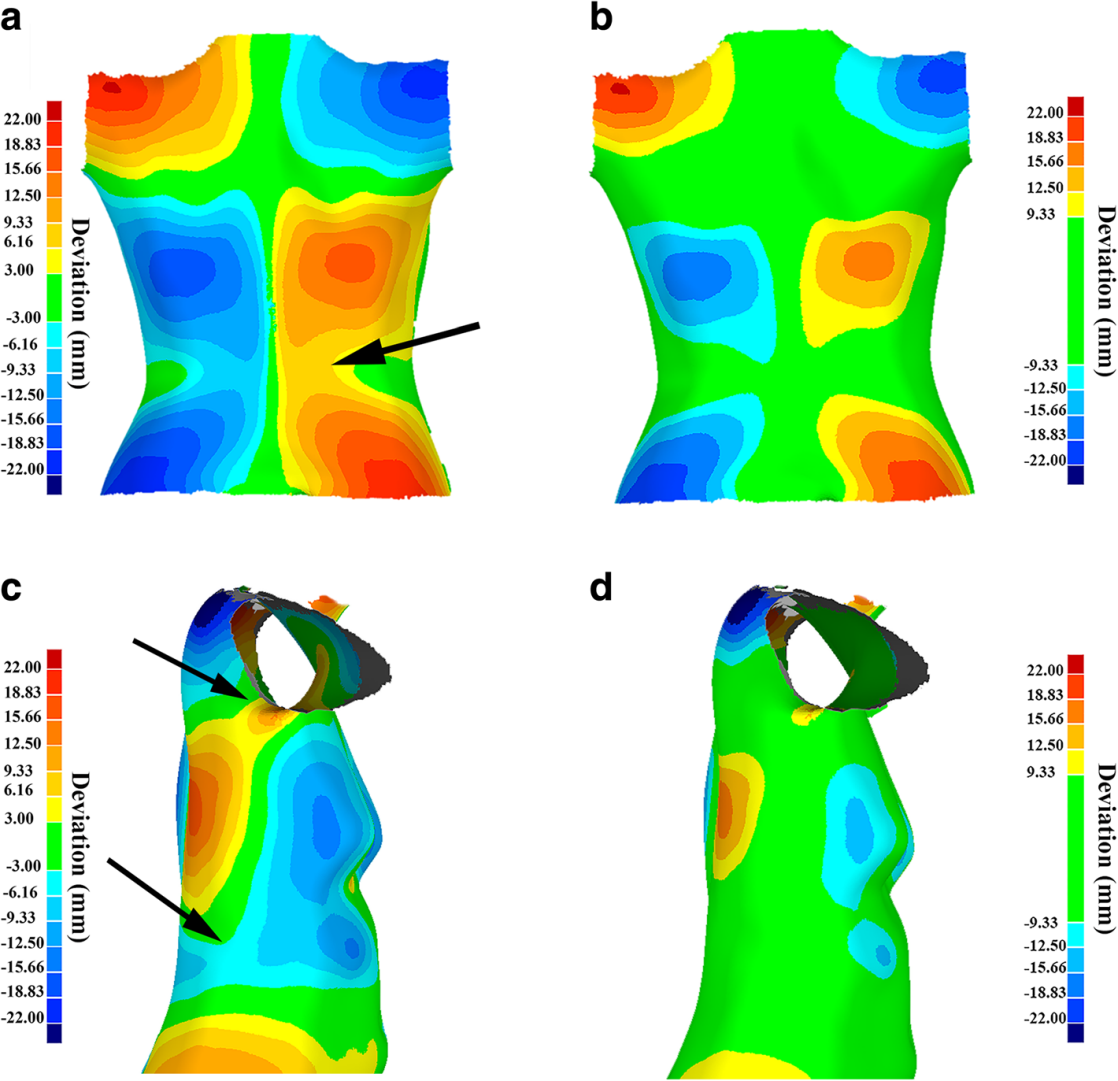
**a**, **c** the deviation patches of two torsos analyzed by Komeili et al. [12], in which the 3 mm deviation was defined as the threshold between normal and deformed area, and **b**, **d** the deviation patches of the same torsos analyzed by the modified ST analysis proposed in this study with the threshold of 9.33 mm. The arrows point to the artifacts in deviation patches, such as continuous deviation patches on the back and side of the torso and deviation patches due to the folded skin near the armpits, which were resolved after using the suggested modifications in this study. The green regions of the torso are considered normal. The blue and red patches represent abnormal protruded and indented regions of the torso, respectively, due to the scoliosis condition

In the study presented by Komeili et al. [12], areas with deviation less than 3 mm were considered normal while greater deviations were considered as a deformation and separated the deviation patches for further analysis. Further application of this proposed ST asymmetry analysis in over 250 AIS patients illustrated that in 30 cases the method failed to either locate the curve properly or to correlate to the corresponding scoliotic curve, and led to misclassification of the AIS severity or progression. For example, in the ST analysis of some patients with a double scoliotic curve, a single isolated deviation patch encompassed the entire back torso (see Fig. 1), and therefore did not reflect the double scoliotic curve in the corresponding radiograph. In some other cases, the deviation patch extended to the anterior part of the torso due to the asymmetry introduced by the breasts or axial rotation of the torso. Folded skin near the armpits and waist also introduced artifact in the ST analysis. The reasons for such lack of correspondence between the surface and the radiographic results were traced to the patch isolating stage where 3 mm was used as a “cut point”. So far, these cases have been manually handled case by case by a scoliosis professional, which can introduce human errors in the measurements and decrease the correlation between the ST parameters and radiograph measurements.

This study aimed to eliminate patch overlaps by enhancing the patch isolation procedure, and to increase the accuracy of the analysis in identifying patients where we could prevent unnecessary X-ray exposure (mild patients and those who did not experience any progression from the last visit). Our hypothesis is that the modifications suggested in this study would isolate the deviation patches without compromising the accuracy of the method in monitoring the scoliotic curve severity or progression.

## Method

### Data collection

Full-torso surface topography scans of 128 AIS patients were collected from the Edmonton Scoliosis Clinic database between October 2009 and 2012. In the cohort, 95 patients (76 females, 19 males) had both baseline and follow-up ST and radiograph scans obtained with an interval of 12 ± 3 months. The inclusion criteria used were patients aged 10 to 18 years old (14.4 ± 1.8 years), with *Cobb angle* greater than 10° (26.5° ± 11.4°) at baseline, with no spine operation.

To develop a classification tree in order to identify the curve severity, 128 baseline radiograph and ST scans were used. In this sample, 99 thoracic-thoracolumbar (T-TL) curves and 98 lumbar (L) curves were measured, with double curves accounting for more curves than the number of patients.

**Fig. 2:**
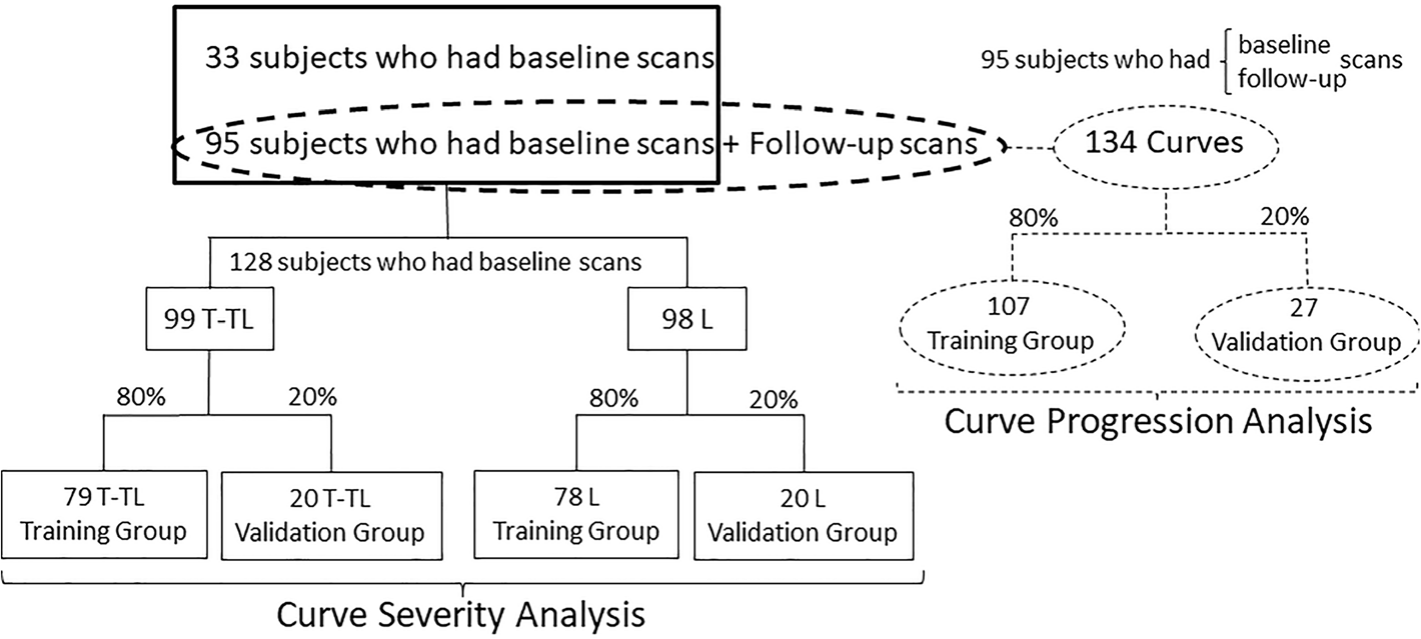
The number and location of curves used in the curve severity and curve progression analyses

To predict the progression of the scoliotic curve, a sample of 95 ST and radiograph scans with corresponding follow-up scans were used, in which the progression of 134 curves in total were analyzed. The data sets were randomly divided into two groups (i.e., the Training group and the Validation group) using common data splitting rules (80/20) [16] as illustrated in Fig. 2. The Training group included 80% of curves and was used in the derivation of the classification tree, whereas the validation group included the other 20% of curves and was used to examine the validity of the obtained classification tree.

### Asymmetry analysis

The ST and radiograph scans were taken on the same day for each patient. The ST data was collected as described previously [12]. Briefly, four VIVID 910 3D laser scanners (KONICA MINOLTA Sensing Inc.) scanned the geometry of the patient’s torso from each side, while the patient was positioned inside a custom designed frame with the torso in its natural posture. The accuracy of the scanning system was 1.8 ± 0.9 mm [17]. The outputs of scanners were four binary files including the spatial locations (i.e., x, y, z coordinates) of the torso surface, and they were imported as inputs to the Geomagic Control software (3D System Corporation, CA, USA) to be merged for the whole torso. Unneeded parts of the scan, namely those for the frame, the head, pants, and arms, were cropped off to isolate the torso [12]. After smoothing the scanning noises, the asymmetry analysis was performed on the torso. The torso was duplicated and reflected along the sagittal plane. Then, the torso and its reflection were aligned by minimizing the sum of squares of distances between these two geometries [18]. The misalignment between the torso and its reflection, resulting from the asymmetry shape of the torso, was measured using the 3D Comparison function and visualized using the contour plot in the Geomagic Control software. In our earlier study [12], the threshold between normal and abnormal deviations was set to 3.0 mm. However, in this study, the threshold, beyond which the asymmetry of the torso was considered scoliotic deformation, was varied from 3 to 10 mm with a 0.33 mm step until the isolated patches matched the curves observed in the radiographs by the clinicians in all subjects. The optimum cut point, which defined the minimum deviation as a scoliotic deformation and avoided patch overlap, was found to be 9.33 mm (Fig. 1). This modification was applied only if the ST scan had a maximum deviation greater than 9.33 mm, otherwise the threshold of 3 mm was used as suggested by Komeili et al. [13, 19]. To measure the ST parameters, the scoliosis deformations were isolated from the other regions creating deformity patches. The macro, that was used by Komeili et al. [12], was modified to isolate the asymmetry patches from the other areas in the deviation contour map in Wolfram Mathematica (Wolfram Research, Inc., Mathematica 8.0.4.0). The process of isolating a deviation patch was as follows:

Step 1- Identify the point with the maximum absolute deviation in the cloud of points and set it as the centre point of a sphere with a radius of 5 mm.
Step 2- Collect all points inside the sphere with the deviation greater than 9.33 mm (the optimum cut point) and include them in the isolated deviation patch.
Step 3- Consider each selected point as the centre of a new sphere.
Step 4- Repeat Step 2 and 3 to progressively expand the boundary of deviation patch.

The maximum deviation (*MaxDev*) used in step 1 and the root mean square (*RMS*) of deviation patches were calculated, respectively, with the following equations:

$$ MaxDev=\mathit{\operatorname{Max}}\left(\left|{Deviation}_i\right|\right)\kern1.2em i=1,2,3,\dots, n $$

$$ RMS=\sqrt{\frac{\sum_{i=1}^n\left({Deviation_i}^2\right)}{n}}\kern1.2em i=1,2,3,\dots, n $$

where, *n* is the number of points representing the torso shape included in a given patch.

Figure 1 shows a contour-plot comparison between the deviation patches of a torso analyzed by Komeili et al. [12] (cut point 3.0 mm) and the deviation patches of the same torso analyzed by the modified ST analysis in this study (cut point 9.33 mm). In the contour plot shown in Fig. 1b, the green represents the area with deviation smaller than 9.33 mm, and shades of blue/red (referred to as deviation patches) indicate the area that protruded/sunken more than 9.33 mm, respectively. The patient’s torso in an ST scan was divided into two parts, namely the lower one-third part was considered the lumbar area (L) and the upper two-third was considered the thoracic / thoraco-lumbar area (T-TL). The asymmetry parameters were assigned to each section, accordingly. The same procedure was repeated for the follow-up ST scans to calculate the progression of *RMS* and *MaxDev*, i.e. *ΔRMS* and *ΔMaxDev*. From the corresponding radiographs, progressions of *Cobb angles* (*ΔCA*) were retrieved from the clinical database. During each patient clinic visit, *Cobb angles* are measured by the clinician and entered in the clinic database along with the end vertebra level and the apex for each curve.

Based on the *Cobb angle*, curve severity is classified into three groups in clinical practice: mild (10° ≤ *Cobb angle* ≤ 25°), moderate (25°< *Cobb angle* ≤ 40°), and severe (40° > *Cobb angle*) [20]. An increase of 5° or more in the *Cobb angle* during consecutive follow-up visits is recognized as a curve progression [21].

### Classification analysis

The classification tree technique implemented in IBM SPSS Statistics 24.0 was employed to build a classification model, using the asymmetry parameters (referred to as independent variables) to classify the curve severity (dependent variable i.e., Mild or Moderate/Severe). On purpose, the criteria in the development of classification tree used in this study placed more weight for the false negative error in the cost function of the classification tree analysis. The underlying rationale for this preference was to prevent classification of moderate or severe scoliotic curves in the mild group as much as possible, which could lead to a late diagnosis or an ineffective treatment of the scoliotic curve in the clinical application.

A separate tree was developed to classify progression (i.e., Progression or Non-progression) of the radiographic curve measurement (dependent variable). Note that the deviation patches in the T-TL and L sections were analyzed together to build the classification tree for categorizing the progression of torso asymmetry. The underlying motivation was the fact that, if a curve progression is predicted by a classification system for a patient, a full vertebra radiograph scan is required regardless of its location and severity.

The classification analysis results were reported in the “Results” section including the tabulated results to show the accuracy, sensitivity, and specificity [19]. In the curve severity prediction, a positive test represents a moderate/severe curve and a negative test represents a mild severity. In the curve progression classification, a positive test represents the curve progression of 5° or more and a negative test represents any curve progression less than 5° (Non-progression).

The classification analysis mentioned above was conducted using the Training group. The obtained classification tree was used to classify the subjects in the Validation group. The resulting accuracy, sensitivity, and specificity of the classification for the Training and Validation groups were compared to assess the validity of the method.

## Results

### Curve severity classification

**Fig. 3:**
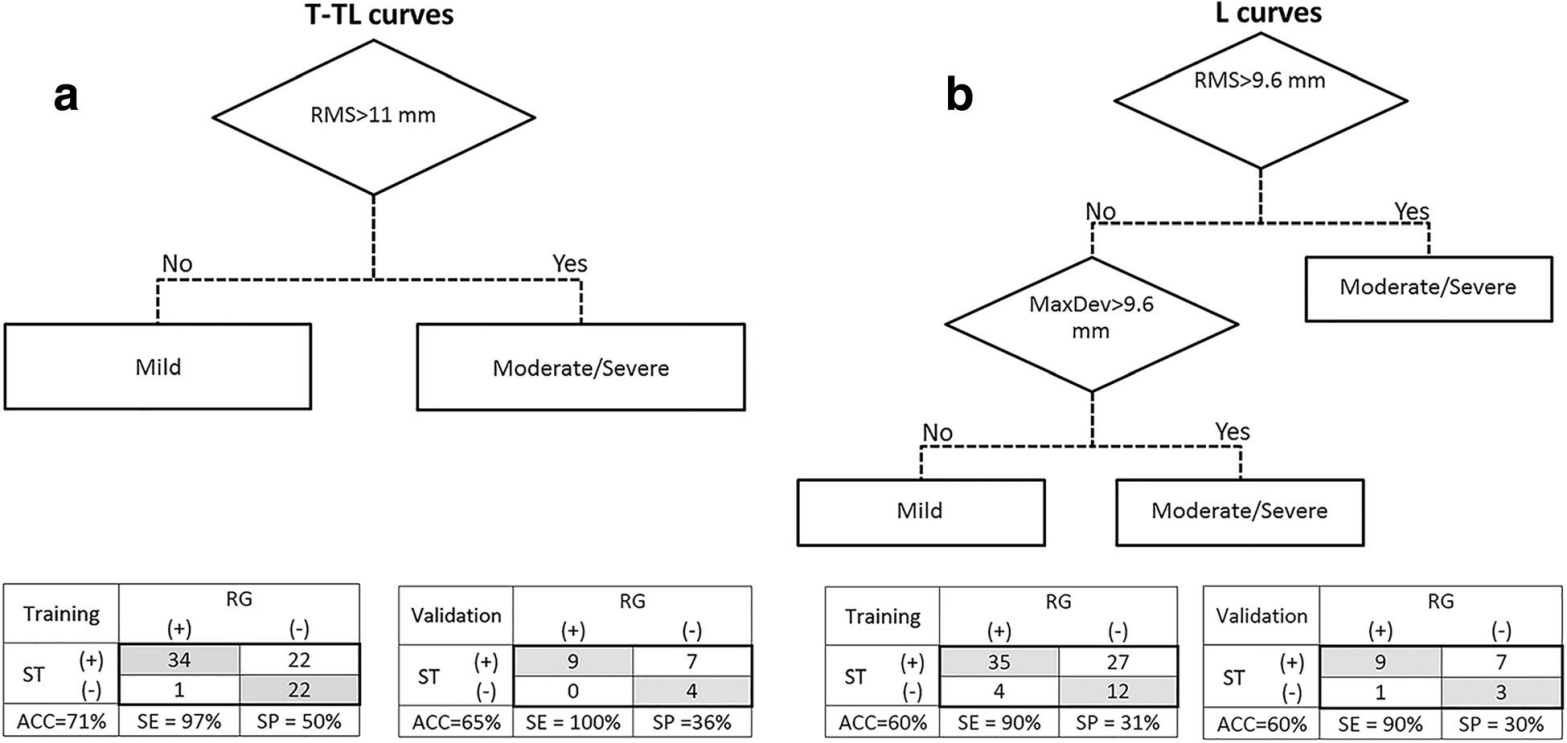
The classification trees and the tabulated accuracy (ACC), sensitivity (SE), and specificity (SP) values for the curve severity classification of (**a**) T-TL curves and (**b**) L curves. The (+) and (−) illustrate the moderate/severe and mild groups, respectively. RG: radiograph, ST: surface topography

None of patients were excluded from the analysis. Figure 3 shows the severity classification trees for T-TL and L curves with the performance indices for the Training and Validation samples. Based on the statistical analysis, the *RMS* was a better independent variable (i.e., predictor) to be used in the classification of T-TL curves, i.e. deviation patches with *RMS* greater than 11 mm in the T-TL section represented a moderate/severe curve regardless of their *MaxDev* value. While the combination of *RMS* and *MaxDev* worked well for identifying the severity of L curves; a deviation patch with *RMS* < 9.6 mm and *MaxDev* < 9.6 mm represented a Mild curve in the L section, otherwise it represented a moderate/severe curve.

Out of 79 T-TL curves in the Training group, there were 35 curves with clinically moderate/severe curves, 34 of which were correctly identified with a sensitivity of 97%. Half of 44 mild curves were correctly classified in the mild group with a specificity of 50%. The majority of patients (22 out of 23), who were classified in the mild T-TL group based on their deviation patches, had truly a *Cobb angle* less than 25° in the corresponding radiographs, resulting in false negative error of only 3%. The overall accuracy of the T-TL curve severity prediction was 71%. The classification of the 20 T-TL curves in the Validation group resulted in similar accuracy and sensitivity, with a maximum difference of ±7%, with respect to the Training group. No moderate/severe thoracic curves were misclassified by the ST analysis in the Validation sample.

In the Training group of 78 deviation patches analyzed for the L section, 39 (50%) had moderate/severe curves based on the *Cobb angle* measurements in radiographs. The ST analysis successfully identified 35 of them in the moderate/severe group, resulting in a sensitivity of 90%. The mild L curves were correctly identified in 31% of cases but only 4 of 39 (11%) moderate/severe curves were missed by the ST analysis. The overall accuracy of the ST analysis in classifying curve severity in the L section was 60%. The classification of the 20 L curves in the Validation group also resulted in the same level of accuracy and sensitivity compared with the Training group. Only one (10%) moderate/severe lumbar curve in the Validation group was misclassified by ST as mild.

**Fig. 4:**
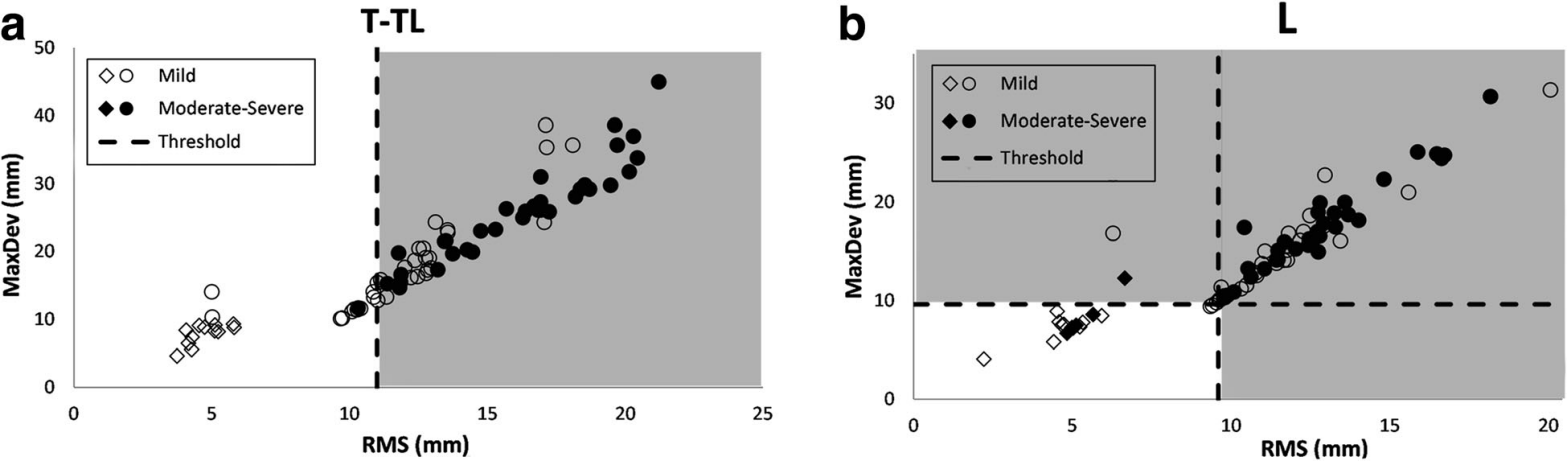
The distribution of *RMS* and *MaxDev* of (**a**) 79 T-TL, and (**b**) 78 L deviation patches and the thresholds for defining the severity of the curves. The shaded area shows the region corresponding to the Moderate-Severe classification. The open and closed symbols represent mild and moderate/severe curves based on the radiograph measurements, respectively. The ♦ and ◊ represent the deviation patches with *MaxDev* < 9.33 mm which were classified using the Komeili et al. [12] classification tree

Figure 4 shows the distribution of *RMS* and *MaxDev* of 79 T-TL and 78 L deviation patches and the thresholds for defining the severity of the curves in the Training group. The ST patches with *MaxDev* less than 9.33 mm were used in the Komeili et al. [12] classification tree to determine the severity of these deviation patches. Only one and four subjects with moderate/severe T-TL and L curves, respectively, were misclassified in the mild region, but their ST parameters were not far below the threshold.

**Fig. 5:**
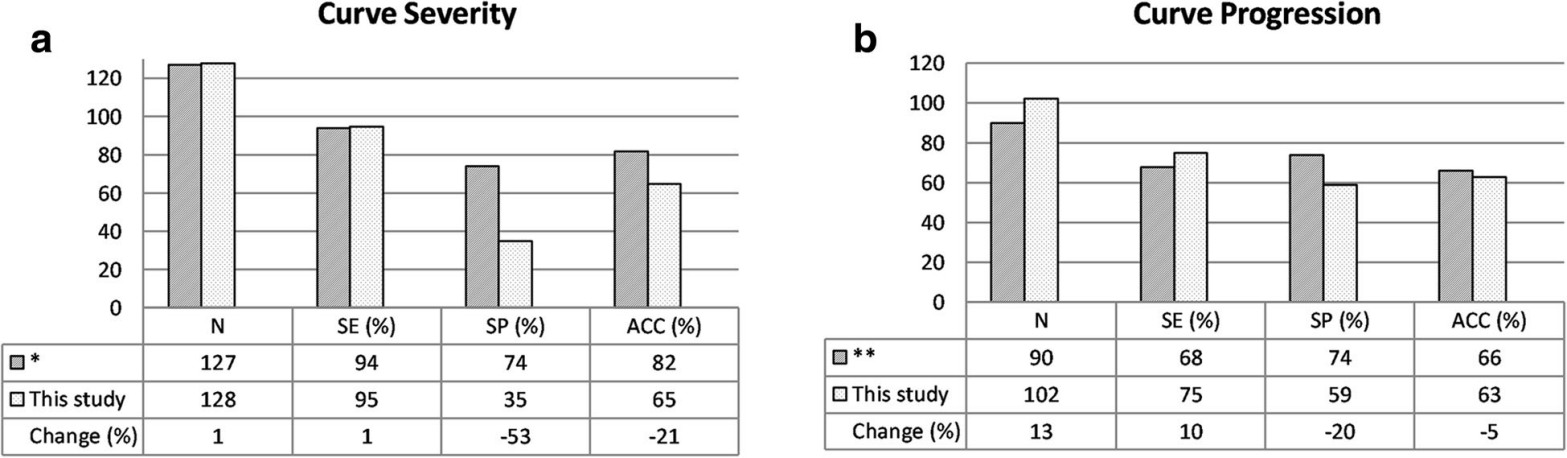
Comparing the performance of the modified ST method in this study for all curves (T-TL and L curves in the Training group and Validation group) with previous studies in predicting the (**a**) severity and (**b**) progression of the scoliotic curves. (*): Komeili et al. [22], (**): Komeili et al. [15]

The performance of the modified ST analysis in identifying the curve severity proposed in this study was compared with our previous work [19, 22] in Fig. 5a. The sensitivity in the prediction of curve severity was as high as the sensitivity in the work presented by Komeili et al. [22].

### Curve progression prediction

**Fig. 6:**
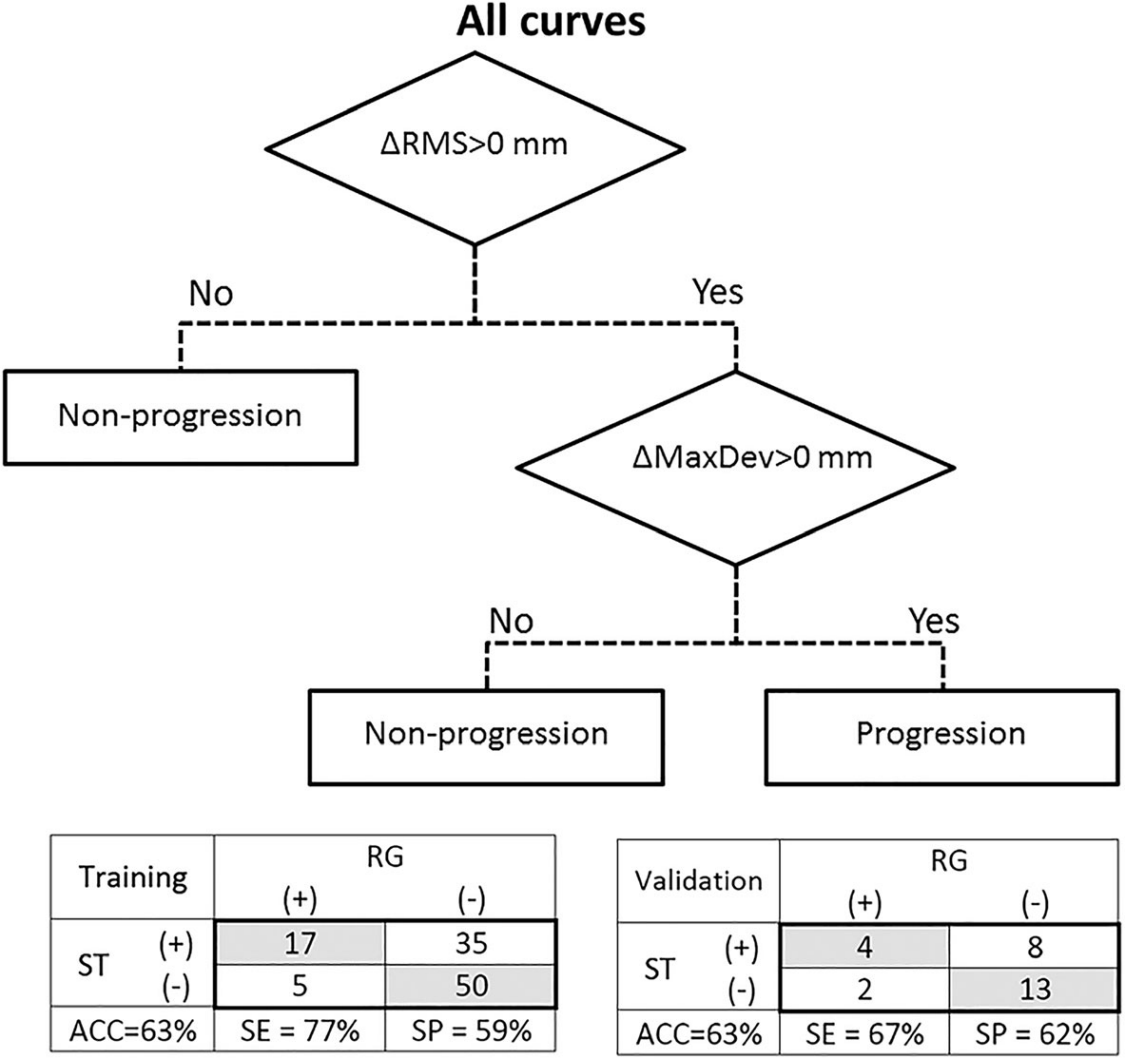
The classification tree for categorizing patients in Progression and Non-progression groups using the Δ*RMS* and Δ*MaxDev* parameters. The (+) and (−) in the tables represent the Progression and Non-progression groups, respectively. ACC: accuracy, SE: sensitivity, SP: specificity, RG: radiograph, ST: surface topography

**Fig. 7:**
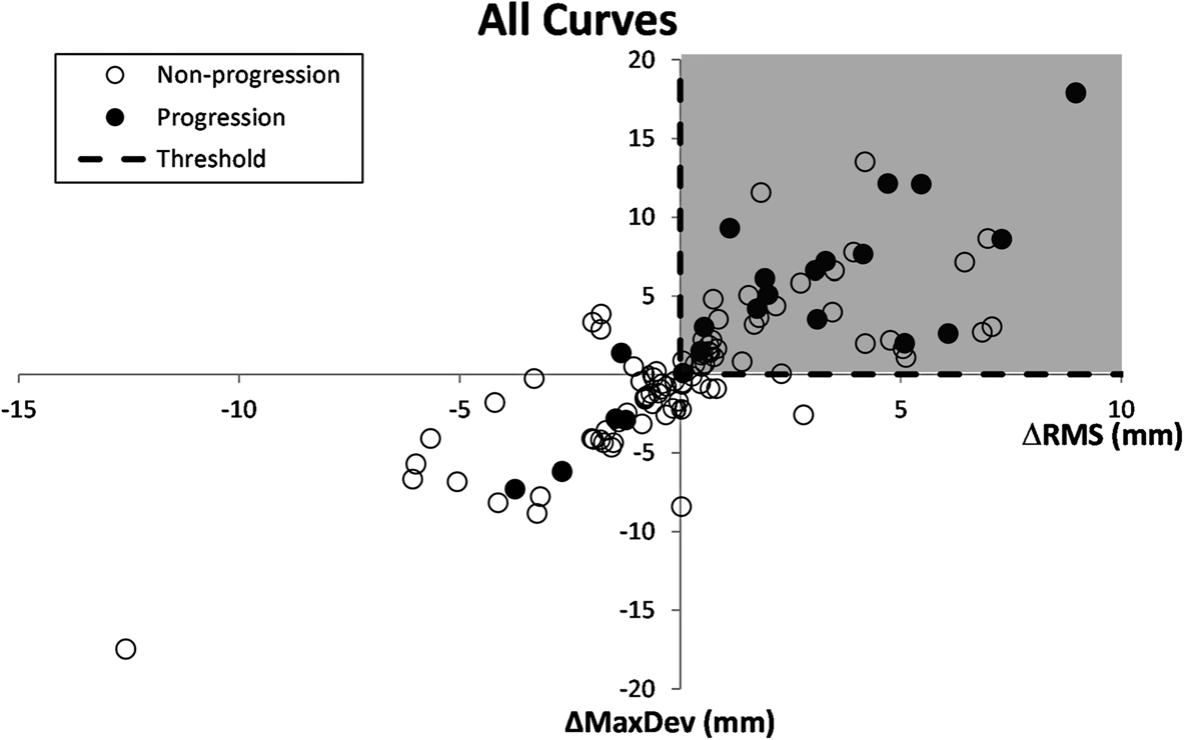
The distribution of Δ*RMS* and Δ*MaxDev* parameters. The threshold of Δ*RMS* and Δ*MaxDev* are shown with the dashed lines. The shaded area shows the region that is considered as the Progression group. The close and open circles represent a progressed (ΔCA ≥ 5°) and Non-progressed (ΔCA < 5°) case based on the radiograph measurements, respectively

Figure 6 shows the classification tree for the prediction of curve progression independent of the curve location, since the T-TL and L curves were mixed in the classification tree analysis. In the classification tree, a positive change of surface topography parameters between two consecutive visits correlated with more than 5° curve progression. In other word, if a curve has positive *MaxDev* and *RMS* changes in the follow-up visit compared with the corresponding values in the baseline assessment, the curve is considered as progression and the patient needs radiography for further assessments. Out of 107 curves in the Training group, 22 curves increased at least by 5°, while there were no clinically important progressions for the other 85 curves. Based on the analysis, 17 out of 22 curves were accurately identified in the progression group (i.e., sensitivity 77%). Five (22%) of the cases with curve progression were missed. The percentage of the non-progressive curves that were detected by the asymmetry analysis was 59%. The diagnostic accuracy was 63%. The classification of deviation patches in the Validation group also resulted in an accuracy of 63% with a sensitivity of 67%, which are close to the corresponding values for the Training group. Figure 7 illustrates the variation of ST parameters in 107 deviation patches analyzed in baseline and follow-up visits, and the thresholds for identifying the scoliotic curves having progressed. There were four patients for whom both ST parameters improved, however their scoliotic curves progressed.

Compared with the sensitivity and accuracy obtained in Komeili et al. [15] study, the sensitivity of the ST method in monitoring curve progression in this study was improved by 10.3% (i.e., from 68 to 75%), while accuracy decreased by a relatively small percentage, 4.5% (i.e., from 66% obtained in Komeili et al. [15] to 63%) (see Fig. 5b).

## Discussion

The markerless asymmetry analysis developed previously [12] showed promising results in identifying curves with progression in the follow-up visits with the potential ability of reducing 44% of radiation exposure in the monitoring of AIS. The research presented in this study aimed at modifying the markerless asymmetry analysis by simplifying the application of the previously developed method while also improving the sensitivity and accuracy of the prediction in identifying the moderate/severe curves and the progression of the scoliotic curve. While the need for manual handling of deviation patches was resolved and the sensitivity in identifying the progressed curves improved by 10%, the overall accuracy of the method decreased by 5%. As a result, the applied modifications on the original method resulted in more conservative monitoring and curve severity assessment of scoliosis where fewer cases of moderate/severe curves and progression cases were missed (see Fig. 5).

In this study, the optimum threshold of deviation for isolating the deviation patches was found to be 9.33 mm. The isolated deviation patches were generally smaller in size than those obtained in Komeili et al. [13, 19] (see Fig. 1). The modified code successfully prevented the extensions of isolated deviation patch to the anterior section of the torso and around the armpits, and clearly separated the boundaries between the deviation patches in ST scans of patients with a double or triple scoliotic curves shown in Fig. 1. Moreover, we expect that the reliability of the method in repeating the analysis would increase by automating the patch isolation step and avoiding human intervention in the ST analysis process.

Accuracies of 65 and 60% were obtained in categorizing the severity of T-TL and L curves in the Validation cohort, respectively as show in Fig. 3. This level of accuracy was approximately the same as the accuracy obtained for the Training group, indicating repeatability of the classification model in the prediction of T-TL curves severity.

The distribution of *MaxDev* and *RMS* parameters of subjects in the Training group in Fig. 4 illustrated that the correlation of ST parameters with the curve severity in the T-TL region was better than in the L region, which is because of the fact that the soft tissue in the L area dampens the deformation of the vertebrae and might soften the effect of the curve in the surface of the torso, while for T-TL curves, the curvature of the vertebrae is transferred to the back surface of the torso through the rib cage and results in prominent deformation [16]. All ST scans with *MaxDev* less than 9.33 mm in Fig. 4, which were denoted by ◊ or ♦, had *Cobb angle* less than 15 degrees and were correctly classified using the original method developed by Komeili et al. [12].

The monitoring of T-TL and L curves progression were analyzed separately (results are not shown) and no difference in the sensitivity, specificity, and accuracy was obtained if T-TL and L curves were analyzed in one group. Therefore, for the monitoring of scoliotic curve, T-TL and L curves were mixed and analyzed using only one classification tree which is likely more practical clinically (Fig. 6). The variation of ST parameters in this study was correlated with the progression of the *Cobb angle* measured in radiographs with a sensitivity of 67% for the Validation group in Fig. 6. However, some negative changes in *ΔRMS* and *ΔMaxDev* were obtained for patients with a positive ΔCA in Fig. 7. The proposed threshold of 9.33 mm for isolating the deviation patches in this study resulted in smaller accuracy and specificity in monitoring curve progression specifically for patients with mild and moderate curves in the baseline. Excluding areas with the deviation less than 9.33 mm from the deviation patches provided less information about the torso shape for monitoring the deformities over time. Therefore, any change in the areas with the deviation less than 9.33 mm over time was not included in the analysis, which led to the misclassification of multiple curves especially in the patients with moderate scoliotic curves, where relatively small portion of torso had deviations greater than 9.33 mm (see Fig. 7). Our results showed that curve progression monitoring appears to be 100% (4/4) accurate for patients with severe scoliotic curve in baseline who progressed in the follow-up scans (tabulated results are not shown). Therefore, monitoring mild and moderate scoliotic curves that present smaller asymmetry on the torso surface with respect to the severe curves, using the modified asymmetry analysis method in this study involves a higher risk of false positive. The case-by-case investigation of patients who were incorrectly identified in the Progression group (false positive) showed that 55% of the population had truly a larger *Cobb angle* in the follow-up radiographs compared to the baseline. However, the positive increase of *Cobb angle* was not significant enough to be considered as a progression, i.e., ΔCA in the range of 0–5 degrees. Although a follow-up radiograph is not necessary for this group of patients, considering the error in *Cobb angle* measurements and the small degree of progression in many of the participants might sufficiently justify taking a radiograph in the follow-up.

The overall sensitivities in identifying curve severity in this study were similar to the corresponding values in Komeili et al. [22], 95 and 94%, respectively (Fig. 5). The reduction in the overall accuracy (i.e. 82 to 65%) in identifying the severity of the curve using ST parameters in this study with respect to Komeili et al. [22] was caused by a larger false positive error rather than false negative error, i.e. classifying a considerable number of the deviation patches of patients with mild scoliotic curves in the moderate/severe group. One third of the misclassified mild curves had a *Cobb angle* in the range of 20–25 degrees, which is on the margin of the mild-moderate severity definition used in the scoliosis clinic. It should be noted that, there is a ±5° inter- and intra-observer error in the measurements of *Cobb angle* [23, 24], therefore there is a possibility that some of the misclassified mild curves with *Cobb angle* in the range of 20–25 degrees could have been diagnosed as moderate if the radiograph assessment had been repeated.

The overall sensitivity of 95% in Fig. 5 indicates a high ability to detect moderate/severe curves which are more susceptible to progress than mild curves and thus do require further attention. Taking full torso radiograph in the follow-up visits is the standard for all patients at the scoliosis clinics, hence, subjects whose classification was false positive would not get more radiographs than others without ST assessment, and we would still prevent radiograph exposure for some truly negative cases. Komeili et al. [15] reported a predicted 44% reduction in the radiograph acquisition if they combined the ST analysis with the radiograph assessment in the monitoring of curve progression. Because of the slightly lower accuracy observed in this study in screening for curve progression, we estimate a 5% lower reduction of the need for radiograph compared with the work of Komeili et al. [15]. Nevertheless, the asymmetry analysis improvements suggested in this study would result to a more conservative monitoring. Because of the higher sensitivity obtained (Fig. 5b) a lower number of patients would suffer from missing the opportunity of an early diagnosis of curve progression by using our improved ST analysis.

We had enough data for statistical analyses, however we have to acknowledge that only 39 patients in the Training group had curve progression, which may not represent the diversity of scoliotic curve types, such as single and double curves. Another limitation of our study is exclusion of trunk axial rotation in our predictions, which is an important piece of information for clinicians in prescribing patient specific braces. It may be possible to improve the accuracy of curve progression diagnosis if more ST parameters, such as curvature of back valley [25] and trunk rotation [26], were combined with the *RMS* and *MaxDev* parameters included in this study. The strong correlation between deviation patches and spinal curve location can also provide good information about the kyphosis and lordosis angles obtained from radiographs, which, however, may not be necessary if the proposed method is used only in scoliosis cases clinics. Our asymmetry analysis is compatible with the ST database of those clinics that do not use the same acquisition system in capturing the full torso geometry as we do. The markerless feature of the asymmetry analysis reduced the common limitations of ST methods, such as dependency of the method to specific local marker-based measurements, and data acquisition technique. Having the 3D geometry of the torso, in normal posture, is the only required condition for using this method. Our strategy in classifying curve severity and curve progression could also be followed in other ST classification systems, in which the cosmetic parameters of the torso are correlated to the scoliotic curve characteristics. Our conservative approach in minimizing the number of false progression and false moderate/severe in the classification system may result in a lower overall accuracy, however it would reduce the risk of missing a progressed or moderate/severe curve, which is the main concern in replacing radiographs with ST assessments in scoliosis clinics.

## Conclusion

The modified thresholds used to define asymmetry patches successfully separated the deviation patches in the upper two-third (T-TL) section from the lower one-third part (Lumbar) section and eliminated the manual work, which was previously necessary for isolating the deviation patches in some patients. The modified thresholds used in this study, allow automation of the analysis and led to the same level of sensitivity in identifying the curve severity as in our previous work. Similarly, the modified analysis led to similar sensitivity for monitoring the progression of the scoliotic curve as our previous work. Despite the novel method being unfortunately associated with a higher risk of misclassification of cases with no progression, significant numbers of patients (approximately 39%) may be able to avoid radiographs confident that their curves did not progress.

## Abbreviations

2D: Two Dimension
3D: Three Dimension
ACC: Accuracy
AIS: Adolescent Idiopathic Scoliosis
L: Lumbar
MaxDev: Maximum deviation of the colour patch
RMS: Root Mean Square of distances between matched points
SE: Sensitivity
SP: Specificity
ST: Surface Topography
T-TL: Thoracic - Thoracolumbar
ΔCA: Change of *Cobb angle*
ΔMaxDev: Change in maximum deviation of the colour patch
ΔRMS: Change in root mean square of the colour patch deviation
